# The Significance of Crescents on the Clinical Features and Outcomes of Primary Immunoglobin A Nephropathy

**DOI:** 10.3389/fmed.2022.864667

**Published:** 2022-06-29

**Authors:** Yongjing Du, Shasha Chen, Fengping Wang, Ping Zhang, Mijia Liu, Chi Liu, Xiang Zhong, Jianhua Qin, Guisen Li, Wei Wang

**Affiliations:** ^1^Department of Nephrology, Institute of Nephrology, Sichuan Academy of Medical Sciences & Sichuan Provincial People’s Hospital, Chengdu, China; ^2^Department of Nephrology, Chengdu Second People’s Hospital, Chengdu, China; ^3^Department of Nephrology, Affiliated Hospital of Southwest Medical University, Luzhou, China

**Keywords:** crescent, IgA nephropathy, clinical features, prognosis, ESKD

## Abstract

**Background:**

It is still controversial whether the proportion of crescents below 50% can be an independent predictive risk factor for poor prognosis in IgAN patients. We reported the significance of different proportions of crescents on the clinical features and the cut-off value of crescents in predicting the occurrence of end-stage kidney disease (ESKD) in patients with IgAN.

**Methods:**

We retrospectively analyzed biopsy-proven primary IgAN patients in Sichuan Provincial People’s Hospital from 2007 to 2019. The patients were divided into 5 groups on the basis of crescent proportion as follows: 0 (*n* = 647), < 10% (*n* = 221), 10 to 24% (*n* = 272), 25 to 49% (*n* = 80), and ≥50% (*n* = 22). The primary endpoint was defined as ESKD, and the secondary endpoint was the combined renal endpoint (≥50% reduction in eGFR or ESKD). A validation cohort of 346 patients were enrolled from Affiliated Hospital of Southwest Medical University. Cox regression model and Kaplan-Meier survival analysis were performed.

**Results:**

A total of 1242 eligible patients with biopsy-proven IgAN were recorded in the database, compared with the non-crescent group, patients in the crescent group had lower levels of hemoglobin (Hb) and albumin (Alb), higher levels of blood urea nitrogen (BUN), 24h urinary protein and hematuria, a higher proportion of mesangial hypercellularity (M1), endocapillary hypercellularity (E1), segmental glomerulosclerosis (S1), and tubular atrophy/interstitial fibrosis (T1/T2) (*p* < 0.05). A higher crescent proportion was associated with lower levels of Hb, ALB, eGFR and serum IgG (*p* < 0.05), higher levels of SCr, BUN, increasing amounts of 24 h urinary protein, increasing proportion of M1 and E1, and increasing severity of interstitial inflammatory infiltration. During the median follow-up of 43 months (range 6-151), 63 individuals (7.0%) reached the primary outcome of ESKD and 99 patients (11.1%) reached the combined renal endpoint. 34(7.5%), 21 (13.3%), 24(12.2%), 14(21.5%) and 6(31.6%) patients reached the combined renal endpoint in the above five groups in crescents 0, <10%, 10∼24%, 25∼49% and ≥50%, respectively. A total of 274(62.6%) cases in the crescent group and 254 (55.7%) cases in the non-crescent group received immunosuppressive therapy. Multivariate Cox regression showed that crescents ≥50% was an independent risk factor for the progression of ESKD (*p* = 0.003) and crescents ≥25% was an independent risk factor for the combined renal endpoint(*p* < 0.001). The receiver operating characteristic curve showed that IgAN patients with crescents ≥43.7% had a higher risk of ESKD, even with immunosuppressants (Sensitivity = 75.7%,specificity = 89.6%,*p* < 0.001). This discovery cohort and the validation cohort further confirmed that patients with crescents <43.7% had better renal prognosis than those with crescents ≥43.7% in the whole group and those with immunosuppressants (*p* < 0.001).

**Conclusion:**

IgAN patients with crescents had more severe clinicopathological features and poorer prognosis. Crescents ≥50% was an independent risk factor for the progression of ESKD and crescents ≥25% was an independent risk factor for ≥50% reduction in eGFR or ESKD in treated and untreated IgAN patients. Crescents ≥43.7% was an independent risk factor for ESKD in those with immunosuppressants.

## Background

Immunoglobin A nephropathy (IgAN) is the most common primary glomerulonephritis in the world. There is considerable heterogeneity in clinicopathological features, rate of disease progression, and prognosis of different IgAN patients. The crescent formation is a common histopathological finding, occurring in approximately 20-60% of IgAN patients. In the original Oxford classification and validation studies for IgAN, the presence or absence of cellular/fibrocellular crescents was not included and crescents also were not considered as a significant predictive and prognostic factor (1). But there were some limitations to these studies, in which individuals with eGFR <30 ml/min/1.73 m^2^ or the rapid progression to end-stage kidney disease (ESKD) were excluded. However, other studies with less restrictive inclusion criteria found a linear relationship between the proportion of crescents and the proportion of IgAN patients with composite end-point events and crescents crescentic were independently related to renal survival. Based on these findings, crescents have been added to Oxford classification, updating to the MEST-C scores (2). According to the revised Oxford Classification, the crescent-score was defined as C0 (no crescents), C1 (crescents in at least 1 but < 25% of glomeruli), or C2 (crescents in at least 25% of glomeruli). There were 61% of patients with crescents <10%, which a multicenter study reported among IgAN patients with crescents (3). Similarly, another study found pathological findings in IgAN patients with the initial eGFR <30 ml/min/1.73 m^2^ had a median crescent of 10% (4). At present, it is widely recognized that crescentic IgAN (crescents involving more than 50% of glomeruli) is a risk factor for the prognosis of patients with IgAN and patients should receive intensive treatment, such as steroids or other immunosuppressive according to the origin Kidney Disease: Improving Global Outcomes (KDIGO) clinical guideline released in 2012 (5). Nevertheless, the prognosis and treatment of patients with crescents <50% is still uncertain (4, 6–14). The above contradictory conclusions suggest that the differences in race, research objects and the inclusion criteria may affect the predictive value of crescents in IgAN. More importantly, the cut-off value of crescents in predicting the occurrence of ESKD in patients with IgAN is also currently inconclusive.

Therefore, our study assessed the value of different proportions of crescents by comparing the clinicopathological features and prognosis of IgAN patients. Furthermore, we also explored the impact of immunosuppressive therapy on the prognosis of IgAN patients with crescents.

## Materials and Methods

### Study Design

#### Patients

This is a single-center retrospective cohort study. Our research protocol was approved by the ethics committee of Sichuan Provincial People’s Hospital. There were 1242 biopsy-proven primary IgAN patients in Sichuan Provincial People’s Hospital from 2007 to 2019. The inclusion criteria were biopsy-proven primary IgAN and age ≥14 years old. Individuals with the number of glomeruli in renal biopsy less than 8, repeating renal biopsy, secondary IgAN caused by Henoch–Schönlein purpura, Sjogren’s syndrome or systemic lupus erythematosus and so on, loss of complete medical records, less than 6 months of follow-up were excluded. The end-point follow-up time was October 1, 2020. Subjects were divided into the crescent group and the non-crescent group based on the presence or absence of crescents. The crescent group was further divided into 4 subgroups based on the proportion of crescents involving glomeruli: <10%, 10∼24%, 25∼49% and ≥50%. To determine the risk factors for ESRD and test the prediction model of IgAN, we further included a validation cohort of 346 patients from Affiliated Hospital of Southwest Medical University (validation cohort; *n* = 346) during 2010 to 2019. The inclusion and exclusion criteria is consistent with our research.

#### Clinical and Laboratory Data at Biopsy

We collected data on individuals’ clinicopathological characteristics at the time of renal biopsy and during follow-up, including blood pressure (BP), serum creatinine (Scr), albumin (Alb), estimated glomerular filtration rate (eGFR), 24 h urine protein, urine red blood cell counts, the medication regimen and so on.

#### Renal Pathological Data at Biopsy

Renal biopsy samples from all patients were reviewed and scored by three experienced renal pathologists. The updated Oxford Classification (MEST-C) was used in this study (mesangial hypercellularity (M0/M1, <or equal to >50% of glomeruli with >4 mesangial cells/area); endocapillary hypercellularity (E0/E1, present/absent); segmental glomerulosclerosis (S0/S1, present/absent); tubular atrophy/interstitial fibrosis (T0/T1/T2, <25%, 25–50%, >50%) (1). A crescent was defined as extra-capillary lesions involving 25% of the glomerular circumference. The proportion of crescents (regardless of the component) was calculated according to the number of crescent affected glomeruli divided by the total number of glomeruli; cellular/fibrocellular/fibrous crescents were considered a component ratio.

#### Treatment and Renal Prognosis

During follow-up, subjects were divided into subgroups based on whether receiving immunosuppressive therapy: the immunosuppressive treatment group and the non-immunosuppressive treatment group. The primary outcome was ESKD, and the combined event was defined as either ESKD or ≥50% decline in eGFR.

### Definitions

Estimated glomerular filtration rate was calculated by Chronic Kidney Disease Epidemiology Collaboration (CKD-EPI). Mean arterial pressure was calculated as 1/3 systolic blood pressure + 2/3 diastolic blood pressure. Immunosuppressive therapy was defined as the use of any immunosuppressive agent, including glucocorticoid (GC), cyclophosphamide (CTX), mycophenolate mofetil (MMF), leflunomide (LEF), cyclosporin A (CsA), tacrolimus (FK506) or hydroxychloroquine (HCQ) and so on, regardless of drug dose and duration (15). Renin-angiotensin-aldosterone system inhibitor (RASi) was defined as any angiotensin-converting enzyme inhibitors (ACEI) or angiotensin II receptor blockers (ARB). ESKD was defined as eGFR <15 ml/min/1.73m2 or the initiation of renal replacement therapy or renal transplantation.

### Statistical Analyses

SPSS 18.0 software was used for data analysis. Continuous variables that followed a normal distribution were expressed as mean ± standard deviation and compared using the *t-test*. Non-parametric variables were expressed as medians (interquartile ranges) and compared using the Mann–Whitney *U* test. Categorical variables were expressed as frequency (percentage) and compared using the chi-square test.

The cumulative survival rate was calculated by the Kaplan-Meier method. Univariate and multivariate Cox regression models were performed to analyze the prognosis of patients with different proportions of crescents by *p*-value [hazard ratio (HR) and 95% confidence interval (95% CI)]. The clinical and pathological factors with a *p*-value <0.1 on univariate analysis were included in the multivariable model. A *p*-value of less than 0.05 was regarded as statistically significant. Receiver operating characteristic (ROC) was used to analyze the diagnostic efficacy of crescents to predict the prognosis of IgAN patients treated with immunosuppressive therapy. We used the area under the curve (AUC) to analyze the relationship between the proportion of crescents and the prognosis of IgAN patients treated with immunosuppressive and calculated the cut-off value of crescents in predicting the occurrence of ESKD in patients with IgAN. Cox regression model and Kaplan-Meier survival analysis were performed in the validation cohort.

## Results

### Demographic Characteristics

**FIGURE 1:**
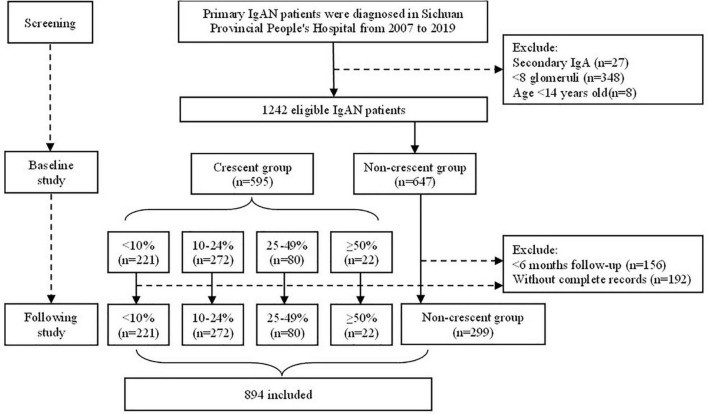
The flow chart of the enrolment of IgAN patients.

A total of 1242 eligible patients with biopsy-proven IgA nephropathy from 2007 to 2019 in Sichuan Provincial People’s Hospital were recorded in the database, of which 595 individuals (47.9%) had different proportions of crescents. The median crescent proportion was 12.5%. The percentage of patients with 0, <10%, 10∼24%, 24∼49% and ≥50% was 52.1% (647 cases), 17.8% (221 cases), 21.9% (272 cases), 6.4% (80 cases) and 1.8% (22 cases), respectively (Supplementary Figure 1). The flow chart of this study was shown in Figure 1.

### Comparisons of Baseline Clinical and Laboratory Variables

Compared with the non-crescent group, the crescent group had lower levels of hemoglobin (Hb) (*p* < 0.001) and albumin (Alb) (*p* < 0.001), higher levels of blood urea nitrogen (BUN) (*p* = 0.021), 24 h urinary protein (*p* < 0.001) and hematuria (*p* < 0.001), a higher ratio of gross hematuria (*p* = 0.03). There was no significant difference in SCr between the two groups, but it showed an upward trend in patients in the crescent group [85.70 (61.91-21.40) μmol/L vs. 80.80 (59.95-115.63) μmol/L, *p* = 0.098] (Supplementary Table 1).

Pathological characteristics of IgAN patients with and without crescents were detailed in Supplementary Table 2. The immunofluorescence findings showed that C3 deposition was more significant in renal tissue of patients with crescents than that without crescents; however, there was no significant difference in IgA, IgG, and IgM deposition between the two groups. The pathological manifestations of patients in the crescent group were severer, including the increase of the proportion of M1, E1, S1, T1 and T2, and the aggravation of interstitial inflammatory infiltration (*p* < 0.05).

We further compared baseline characteristics of four subgroups of IgAN with crescents, as shown in Table 1. A higher crescent proportion was associated with lower levels of Hb, ALB, eGFR and serum IgG (*p* < 0.05), higher levels of SCr, BUN and increasing amounts of 24 h urinary protein. With the increasing ratio of crescents, patients were more prone to acute kidney injury (AKI), especially in the group with crescents ≥50, which was significantly higher than that in other groups.

**TABLE 1:** Baseline characteristics of IgAN patients with different proportions of crescents.

	<10% (*n* = 221)	10∼24% (*n* = 272)	25∼49% (*n* = 80)	≥ 50% (*n* = 22)	*P-value*
Gender, Male/Female^c^	95/126	118/154	34/46	12/10	0.770
Age, Year^a^	33.03 ± 11.73	34.97 ± 12.58	36.59 ± 14.83	33.86 ± 14.48	0.138
MAP, mmHg^a^	94.93 ± 11.91	94.88 ± 12.64	95.55 ± 12.18	98.86 ± 14.18	0.519
Hb, g/L^a^	132.20 ± 17.91	127.37 ± 19.44	124.55 ± 23.43	119.32 ± 19.65	0.001*
Cr, μmol/L	76.60 (60.30-104.75)	84.50 (61.00-122.98)	101.75 (73.30-134.80)	154.50 (91.03-261.00)	<0.001**
eGFR, ml/min/1.73m^2b^	101.41 (72.75-131.16)	88.98 (58.98-124.05)	68.32 (42.51-107.98)	41.53 (23.34-91.28)	<0.001**
UA, μmol/L^a^	369.25 ± 111.25	381.57 ± 113.96	376.09 ± 106.40	376.11 ± 104.51	0.689
BUN, mmol/L^a^	5.71 ± 2.24	6.57 ± 2.99	7.36 ± 3.77	9.05 ± 5.68	<0.001**
Alb, g/L^b^	40.75 (36.53-43.90)	38.60 (34.05-42.75)	34.40 (27.78-40.15)	26.85 (24.38-33.88)	<0.001**
Gross hematuria, n (%)^c^	75(33.9)	79(29.0)	29(36.3)	8(36.4)	0.432
Urinary RBC, n (%)^c^					<0.001**
1 +	36(16.3)	41(15.1)	9(11.3)	1(4.5)	
2 +	63(28.5)	76(27.9)	15(18.8)	5(22.7)	
3 +	91(41.2)	147(54.0)	53(66.3)	11(50.0)	
24h urinary protein, g/24h^b^	1.28 (0.35-2.04)	1.59 (1.00-3.10)	2.55 (1.60-4.16)	3.59 (0.64-2.32)	<0.001**
Serum C3, g/L^a^	1.08 ± 0.25	1.11 ± 0.37	1.05 ± 0.26	1.14 ± 0.44	0.41
Serum C4, g/L^a^	0.27 ± 0.09	0.27 ± 0.09	0.28 ± 0.11	0.28 ± 0.10	0.795
Serum IgG, g/L^a^	10.47 ± 3.59	9.79 ± 3.55	9.41 ± 5.41	6.14 ± 2.55	<0.001**
Serum IgA, g/L^a^	3.03 ± 1.15	3.02 ± 1.22	2.95 ± 1.41	2.35 ± 1.02	0.166
AKI, n (%)^c^	6(2.7)	4(1.5)	6(7.5)	6(27.3)	<0.001**
CKD, n (%)^c^					<0.001**
CKD1	135(61.1)	142(52.2)	28(35.0)	5(22.7)	
CKD2	45(20.4)	54(19.9)	22(27.5)	3(13.6)	
CKD3	20(9.0)	47(17.3)	9(11.3)	4(18.2)	
CKD4	7(3.2)	17(6.3)	10(12.5)	1(4.5)	
CKD5	8(3.6)	8(2.9)	5(6.3)	3(13.6)	

MAP, mean arterial pressure; Hb, hemoglobin; Cr, creatinine; eGFR, estimated glomerular filtration rate; UA, uric acid; BUN, blood urea nitrogen; Alb, albumin; AKI, acute kidney injury; CKD, chronic kidney disease. ^a^Normal distribution was expressed as mean ± standard deviation and compared using the t-test. ^b^Non-parametric variables were expressed as medians (interquartile ranges) and compared using the Mann–Whitney U test. ^c^Categorical variables were expressed as frequency (percentage) and compared using the chi-square test *p < 0.05; **p < 0.001.

There was no significant difference in the expression of IgA, IgG, IgM, and C3 in renal tissue between different proportions of crescent subgroups (Table 2). Light microscopy findings showed that a higher crescent proportion was associated with increasing proportion of M1, E1, and increasing severity of interstitial inflammatory infiltration.

**TABLE 2:** Pathological characteristics of IgAN patients with different proportions of crescents.

	<10% (n = 221)	10∼24% (n = 272)	25∼49% (n = 80)	≥50% (n = 22)	*P-value*
IgA, n (%)^c^					0.636
1∼2 +	53(24.0)	66(24.3)	26(32.5)	7(31.8)	
3 +	158(71.5)	196(72.1)	50(62.5)	14(63.6)	
4 +	10(4.5)	10(3.7)	4(5.0)	1(4.5)	
IgG, n (%)^c^					0.599
Negative	172(77.8)	224(82.4)	65(81.3)	16(72.7)	
1 +	41(18.6)	39(14.3)	13(16.3)	4(18.2)	
2∼3 +	8(3.6)	9(3.3)	2(2.5)	2(9.1)	
IgM, n (%)^c^					0.785
Negative	53(24.0)	59(21.7)	17(21.3)	6(27.3)	
1 +	125(56.6)	157(57.7)	41(51.3)	11(50.0)	
2∼3 +	43(19.5)	56(20.6)	22(27.5)	5(22.7)	
C3, n (%)^c^					0.888
Negative ∼1 +	49(22.2)	56(20.6)	20(25.0)	4(18.2)	
2 +	114(51.6)	138(50.7)	36(45.0)	10(45.5)	
3∼4 +	58(26.2)	78(28.7)	24(30.0)	8(36.4)	
M1, n (%)^c^	85(38.5)	134(49.3)	56(70.0)	16(72.7)	<0.001**
E1, n (%)^c^	50(22.6)	96(35.3)	35(43.8)	9(40.9)	0.001*
S1, n (%)^c^	123(55.7)	151(55.5)	42(52.5)	8(36.4)	0.358
T1, n (%)^c^	20(9.0)	24(8.8)	8(10.0)	5(22.7)	0.225
T2, n (%)^c^	6(2.7)	17(6.3)	5(6.3)	2(9.1)	0.137
Global glomerulosclerosis,%^b^	0(0-11.11)	0(0-13.13)	0(0-13.84)	0(0-8.04)	0.849
Interstitial inflammation, *n* (%)^c^					0.004*
Negative	59(26.7)	53(19.5)	24(30.0)	4(18.2)	
Mild	141(63.8)	190(69.9)	50(62.5)	10(45.5)	
Moderate	14(6.3)	12(4.4)	3(3.8)	4(18.2)	
Severe	7(3.2)	17(6.3)	3(3.8)	4(18.2)	
Cellular crescent, *n* (%)^c^	0(0-50.0)	0(0-50.0)	25.0(0-97.5)	26.8(0-84.7)	0.004*
Fibrous crescent,%^b^	0(0-100.0)	25.0(0-66.7)	25.0(0-50.0)	5.0(0-52.1)	0.604
Fibro-cellular crescent,%^b^	0(0-100.0)	25.0(0-66.7)	25.0(0-50.0)	21.1(0-53.6)	0.503

*The results of IgA, IgG, IgM, and C3 were manifestations of immunofluorescence. Cellular/fibrous/fibrocellular crescents were calculated according to the relative ratio.*

^b^Non-parametric variables were expressed as medians (interquartile ranges) and compared using the Mann–Whitney U test. ^c^Categorical variables were expressed as frequency (percentage) and compared using the chi-square test *p < 0.05; **p < 0.001.

### Immunosuppressive and/or Renin-Angiotensin-Aldosterone System Inhibitor Therapy

A total of 894 cases were included in the following study, among which, there were 254 of 456 cases (55.7%) treated with immunosuppressive therapy in the non-crescent group and 274 of 438 cases (62.6%) received immunosuppressive therapy in the crescent group. With the increasing ratio of crescents, the proportion of patients who received immunosuppressive therapy also increased (*p* < 0.001). In this cohort, we found no significant difference in the proportion of individuals treated with RASi among the groups (Table 3).

**TABLE 3:** During follow-up patients with or without immunosuppression and RASi.

	Crescent group (*n* = 438)				Non-crescent group (*n* = 456)	*P-value*
	
	<10% (*n* = 158)	10∼24% (*n* = 196)	25∼49% (*n* = 65)	≥ 50% (*n* = 19)		
Receiving Immunosuppression, n (%)^c^	80 (50.6)	122 (62.2)	53 (81.5)	19 (100)	254 (55.7)	<0.001**
Receiving RASi, n (%)^c^	137 (86.7)	166 (84.7)	56 (86.2)	13 (68.4)	391(85.7)	0.354

*RASi, renin-angiotensin-aldosterone system inhibitor. ^c^Categorical variables were expressed as frequency (percentage) and compared using the chi-square test.*

***p < 0.001.

### Prognosis

After an average follow-up of 43 months (range 6-151 months), 99 patients (11.1%) reached the combined renal endpoint, of which 63 patients (7.0%) experienced ESKD, 36 patients (4.0%) experienced a ≥50% reduction in eGFR. 21 (13.3%) cases with crescents <10%, 24 (12.2%) cases with crescents 10∼24%, 14 (21.5%) cases with crescents 25∼49% and 6 (31.6%) cases with crescents ≥50% reached combined end point, respectively. In the non-crescent group, 34 (7.5%) cases reached the combined renal endpoint.

**FIGURE 2:**
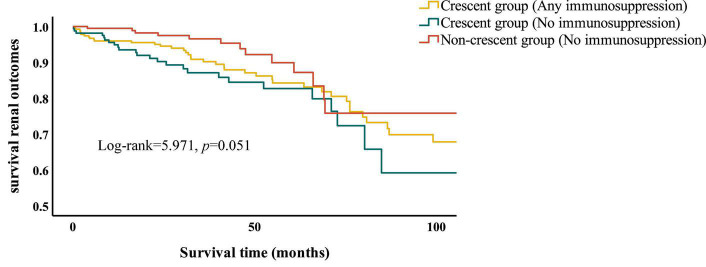
Kaplan Meier survival curve of IgAN patients with or without crescents.

In the discovery cohort, we used univariate Cox regression to analyze the factors affecting renal outcomes in IgAN patients (Supplementary Table 3). The results showed that age, SCr, UA, BUN, 24 h urinary protein, serum IgA, M1, S1, T1, T2, global glomerulosclerosis, crescents and interstitial inflammatory infiltration were related to the development of combined renal endpoint. The above factors were analyzed by multivariate analysis through COX proportional hazard regression model (Table 4). The results showed that MAP, SCr, 24 h urinary protein, serum IgA, T1, T2, crescents ≥50% and global glomerulosclerosis were independent risk factors for ESKD and composite renal outcomes in IgAN patients. We also compared combined events in treated versus untreated individuals using multivariate analysis through COX proportional hazard regression model (Table 5). The results showed that crescent ≥25% was an independent risk factor for combined renal outcomes in IgAN patients with immunosuppressive therapy and any proportion of crescents was an independent risk factor for poor prognosis in IgAN patients. Kaplan Meier survival curve was used to analyze the effect of immunosuppressive therapy on the renal survival rate of IgAN patients with or without crescents (Figure 2). The results showed that, for patients treated with immunosuppressive therapy, the 5-year renal survival rate was 86.2% in the crescent group. For patients without immunosuppressive therapy, there were 84.5 and 92.2% individuals in the crescent group and non-crescent group, respectively. There was no significant difference in renal survival among the three groups. However, for patients without immunosuppressive therapy, the 100-month renal survival rate in the non-crescent group was higher than that in the crescent group [59.2 *vs*. 75.8%, *p* < 0.05].

**TABLE 4:** Multivariate analysis of clinical and pathological factors for the combined events.

Parameters	ESKD			Combined events		
		
	HR	95%CI	*p* Value	HR	95%CI	*P-value*
MAP	1.019	1.002-1.036	0.026*	1.020	1.003-1.037	0.021*
Cr, μmol/L	1.002	1.001-1.004	0.001*	1.001	1.000-1.003	0.045*
24 h urinary protein, g/24h	1.305	1.208-1.410	<0.001**	1.253	1.172-1.341	<0.001**
Serum IgA, g/L	1.424	1.067-1.901	0.016*	1.261	1.023-1.555	0.03*
M1(M0/M1)	1.315	0.620-2.791	0.475	0.775	0.470-1.277	0.317
E1(E0/E1)	1.303	0.638-2.662	0.467	1.255	0.763-2.061	0.371
S1(S0/S1)	0.723	0.321-1.629	0.434	0.945	0.562-1.589	0.832
T1(T0/T1/T2)	7.005	3.012-16.291	<0.001**	7.747	4.314-13.913	<0.001**
**Proportions of crescents**						
None	Reference			Reference		
<10%	2.567	0.956-6.890	0.061	1.695	0.952-3.018	0.073
10∼24%	1.999	0.792-5.046	0.143	1.623	0.919-2.866	0.095
25∼49%	1.675	0.474-5.913	0.423	4.109	2.046-8.254	<0.001**
≥50%	8.652	2.068-36.194	0.003*	5.259	1.971-14.025	0.001*
Global glomerulosclerosis,%	1.019	1.000-1.039	0.047*	1.016	1.001-1.032	0.039*
**Immunosuppressive therapy**						
No Immunosuppression	Reference			Reference		
Any Immunosuppression	0.378	0.189-0.753	0.006*	0.195	0.110-0.346	<0.001**

The clinical and pathological factors with a p-value <0.1 on univariate analysis were included in the multivariable model. MAP, mean arterial pressure; Cr, creatinine *p < 0.05; **p < 0.001.

**TABLE 5:** Analysis of immunosuppressive therapy in subgroups of the crescents group.

Parameters	Untreated with immunosuppression			treated with immunosuppression		
		
	HR	95%CI	*p* Value	HR	95%CI	*P-value*
**Proportions of crescents**						
None	Reference			Reference		
<10%	3.978	1.437-11.011	0.008*	1.653	0.525-5.202	0.39
10∼24%	5.090	1.639-15.809	0.005*	1.633	0.568-4.694	0.362
25∼49%	6.746	1.524-29.873	0.012*	3.944	1.342-11.592	0.013*
≥50%	—	—	—	4.332	1.113-16.867	0.035*

The clinical and pathological factors with a p-value <0.1 on univariate analysis were included in the multivariable model *p < 0.05.

**FIGURE 3:**
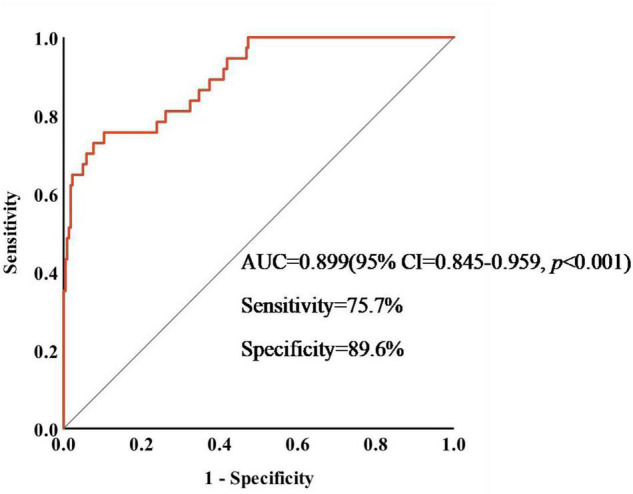
Sensitivity and specificity of crescents in predicting the occurrence of ESKD in IgAN patients.

We drew a ROC curve to analyze the diagnostic efficacy of crescents to predict the prognosis of IgAN patients treated with immunosuppressants (Figure 3). AUC under ROC curve was 0.899 (95% CI = 0.845-0.959, *p* < 0.001). The cut-off value of the proportion of crescents in predicting the occurrence of ESKD in IgAN patients calculated by the Youden Index was 43.7% (Sensitivity = 75.7%,specificity = 89.6%, *p* < 0.001). The prognosis of IgAN patients with crescent ≥43.7% was still poor, even after receiving immunosuppressive therapy.

**FIGURE 4:**
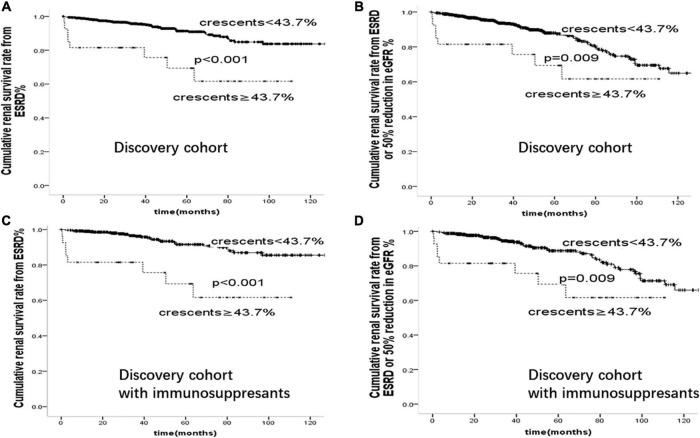
Cumulative renal survival rate from **(A)**: ESRD **(B)**: ESRD or 50% reduction in eGFR in 894 cases of IgAN patients. Cumulative renal survival rate from **(C)**: ESRD **(D)**: ESRD or 50% reduction in eGFR in 529 cases of IgAN patients treated with immunosuppressants.

We further compared the risk of ESKD associated with crescents ≥43.7% in our whole cohort and within subgroups treated with immunosuppressants in Figure 4. Patients with crescents <43.7% had better renal prognosis than those with crescents ≥43.7% (*p* < 0.001), supporting the predictive value of 43.7% in IgA patients with or without immunosuppressive therapy.

### Validation of Prediction Model

**FIGURE 5:**
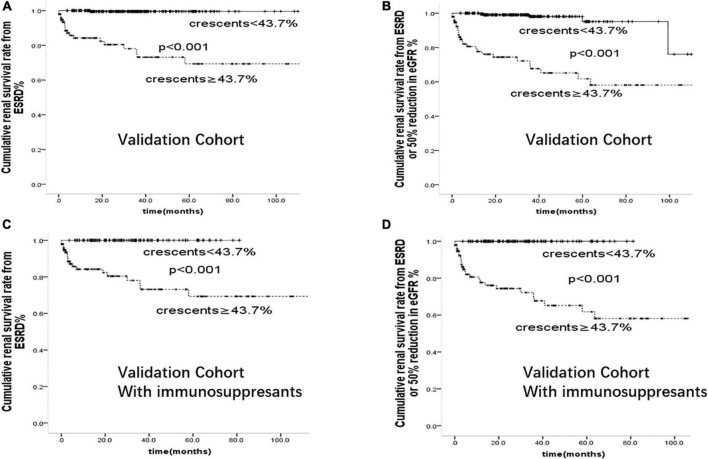
Cumulative renal survival rate from **(A)**: ESRD **(B)**: ESRD or 50% reduction in eGFR in 346 cases of IgAN patients from validation Cohort. Cumulative renal survival rate from **(C)**: ESRD **(D)**: ESRD or 50% reduction in eGFR in 206 cases of IgAN patients treated with immunosuppressants from validation Cohort.

To determine the risk factors and test the prediction model of crescentic IgAN, we further confirmed the above results in a validation cohort of 346 patients from Affiliated Hospital of Southwest Medical University. Comparison of baseline characteristics of IgAN patients in this discovery cohort and validation cohort was shown in Supplementary Table 4. On multivariate cox regression analyses in the validation cohort, glomerular sclerosis (*p* < 0.001), crescents (*p* < 0.001), and serum Creatinine (*p* < 0.001) were risk factors for the development of ESRD. As shown in Figure 5, after adjusted for proteinuria, eGFR, blood pressure, Oxford-MEST score, patients with crescents <43.7% had better renal prognosis than those with crescents ≥43.7% in the whole group and those with immunosuppressants(*p* < 0.001).

## Discussion

Although the origin Oxford classification for IgAN has been extended to the MEST-C scores, the impact of the crescents on the poor outcomes in IgAN patients is still uncertain, especially for the proportion of crescent below 50%, and the threshold of crescent proportion for predicting poor prognosis of IgAN is also controversial. The crescent score wasn’t included in the International IgAN Prediction Tool recommended for quantifying the risk of IgAN progression. Furthermore, a multicentre study from China found that the presence of crescents was not a predictor of poor prognosis in IgAN patients (9), which excluded individuals with eGFR<30 ml/min/1.73m^2^ and 24 h urinary protein <0.5 g/24 h. Therefore, our study aimed to evaluate the value of different proportions of crescents (including crescent below 50%) by comparing the clinicopathological features and prognosis of IgAN patients including those with rapid progression renal function.

The glomerular crescent is a histomorphological indicator of a rupture of glomerular capillaries, which is related to various clinical manifestations, such as blood pressure, SCr, 24-h urinary protein, anemia and so on. Our study confirmed that the baseline clinical and pathological indicators were more severe with the increase of the proportion of crescent, which was similar to the results of other cohort studies (15–17). Crescents formation leads to fading of eGFR by increasing counter pressure and collapse of the glomerular tuft or by obstructing the tubular outflow (18). A multicenter study showed that the blood pressure of IgAN patients in group C2 was higher than that of C0 and C1 (15). However, there was no significant difference in MAP between the -crescent and the none-crescent group, in our study. The possible reason for this result is that we did not exclude patients who had received RASi before enrolment.

Our current report represents the largest cohort to date to investigate the effect of different proportions of crescents on the clinical outcomes of IgAN patients. In our study, after adjusting clinical and pathological factors, we showed crescents ≥50% was associated with the progression of IgAN patients to ESKD, and crescents ≥25% was an independent risk factor for combined renal outcomes, consistent with previous reports. Moreover, we found that among patients without immunosuppressive therapy, there was no significant difference in baseline SCr between patients with crescents and without crescents, but the former’s long-term renal survival rate was significantly lower. The results of another retrospective analysis also supported our conclusions (13). Similarly, this study showed that, after adjusting for age, gender, 24 h urinary protein and baseline SCr, the presence of crescents was independently associated with composite point events (doubling of baseline SCr or ESKD or death). Research at home and abroad shows that the value of crescents in predicting renal function progression is inconsistent in IgAN patients. On the one hand, it is considered that race, geographical distribution, inclusion criteria may affect the predictive value of crescents on the progression of IgAN. On the other hand, calculating the percentage of crescents is not a precise method, because it is affected by the size of the biopsy sample and the number of histologic sections examined (13). In addition, our baseline data analysis showed that the patients with crescents <10% were 37.1%. In fact, it’s easy to ignore the crescent lesion in these patients, especially for those without severe renal function injury. Therefore, clinicians should pay more attention to the crescent lesion, even if the proportion of crescents is less than 10%, and early treatment can effectively delay the progression and improve the prognosis of IgAN.

According to the origin KDIGO clinical guideline released in 2012 (5), IgAN patients with crescentic IgAN were recommended to receive more intensive treatment, such as GC or other immunosuppressive. However, for IgAN patients with crescents < 50%, the specific treatment of these patients was still unclear. Additionally, with reference to the 2021 edition “KDIGO 2021 Clinical Practice Guideline for the Management of Glomerular Diseases,” it pointed out that there was insufficient evidence to make treatment decisions based on the presence and number of crescents in renal biopsy (19). Our study showed that IgAN patients with crescents received more intensive treatment. In these patients, crescents <25% was not an independent risk factor for poor prognosis. However, for IgAN patients without immunosuppressive therapy, any proportion of crescents was a predictor of composite renal outcome. Therefore, immunosuppressive therapy may delay the progression and improve the prognosis of IgAN, which was similar to the conclusion of Haas (3).

This discovery cohort and the validation cohort both confirmed that patients with crescents <43.7% had better renal prognosis than those with crescents 43.7% in the whole group and those with immunosuppressants (*p* < 0.001). Based on the above conclusions, we speculated that immunosuppressive therapy may delay the occurrence of composite renal outcomes, and patients with crescents ≥43.7% may still have a poor prognosis, even with intensive immunosuppressive therapy. Therefore, it’s important to perform a renal biopsy as early as possible in patients with suspected IgA nephropathy.

There were some limitations to this study. Firstly, as a retrospective study, we didn’t describe the kinds of immunosuppressive, drug doses and duration of treatment in detail. Secondly, this was a single-center retrospective observational analysis, it was difficult to control for all factors that may affect renal survivorship. We hope to perform multicenter prospective research with a large sample size to further verify this conclusion.

## Conclusion

Immunoglobin A nephropathy patients with crescents had more severe clinicopathological features and poorer prognosis. Crescents ≥50% was an independent risk factor for the progression of ESKD and crescents ≥25% was an independent risk factor for ≥50% reduction in eGFR or ESKD in treated and untreated IgAN patients. Crescents ≥43.7% was an independent risk factor for ESKD in those with immunosuppressants.

## Data Availability Statement

The original contributions presented in this study are included in the article/Supplementary Material, further inquiries can be directed to the corresponding author/s.

## Ethics Statement

The studies involving human participants were reviewed and approved by Ethics Committee of Sichuan Academy of Medical Science & Sichuan Provincial People’s Hospital. Written informed consent from the participants/patients was not required to participate in this study in accordance with the national legislation and the institutional requirements.

## Author Contributions

YD, SC, WW, PZ, ML, XZ, JQ, and WW: data collection. GL and WW: study design. YD, WW, FW, and ML: statistical analyses. YD, WW, SC, and CL: writing. All authors have read and approved the manuscript.

## Conflict of Interest

The authors declare that the research was conducted in the absence of any commercial or financial relationships that could be construed as a potential conflict of interest.

## Publisher’s Note

All claims expressed in this article are solely those of the authors and do not necessarily represent those of their affiliated organizations, or those of the publisher, the editors and the reviewers. Any product that may be evaluated in this article, or claim that may be made by its manufacturer, is not guaranteed or endorsed by the publisher.
