# Supplementary material for: The Significance of Crescents on the Clinical Features and Outcomes of Primary Immunoglobin A Nephropathy
Source: Front Med (Lausanne). 2022 Jun 29;9:864667. doi: 10.3389/fmed.2022.864667 (PMC9276938; doi:10.3389/fmed.2022.864667)
Supplement: Supplementary file 1 [file Data_Sheet_1.docx]

# Additional files

**Table S1.** Baseline characteristics of IgAN patients with and without crescents

|  | Crescent group  (n=595) | Non-crescent group  (n=647) | *p* Value |
| --- | --- | --- | --- |
| Gender, Male/Female ^c^ | 259/336 | 290/357 | 0.648 |
| Age, Year ^a^ | 34.4±12.7 | 35.4±11.5 | 0.146 |
| MAP, mmHg ^a^ | 95.13±12.36 | 95.46±12.93 | 0.643 |
| Hb, g/L ^a^ | 128.49±19.72 | 135.04±20.27 | <0.001^**^ |
| SCr, μmol/L ^b^ | 85.70(61.9-121.40) | 80.80(59.95-115.63) | 0.098 |
| eGFR, ml/min/1.73m^2 b^ | 90.16(59.00-124.17) | 94.15(59.32-116.36) | 0.354 |
| UA, μmol/L ^a^ | 376.08±111.51 | 376.37±112.49 | 0.964 |
| BUN, mmol/L ^a^ | 6.45±3.09 | 6.08±2.54 | 0.021^*^ |
| Alb, g/L ^b^ | 38.90(33.50-42.70) | 40.65(36.20-43.80) | <0.001^**^ |
| Gross hematuria, n (%) | 191(32.1) | 158(24.4) | 0.03^*^ |
| Urine RBC, n (%) ^c^ |  |  | <0.001^**^ |
| 1+ | 87(14.6) | 142(21.9) |  |
| 2+ | 159(26.7) | 148(22.9) |  |
| 3+ | 302(50.8) | 183(28.3) |  |
| 24h urinary protein, g/24h ^b^ | 1.60(0.93-3.05) | 1.19(0.64-2.32) | <0.001^**^ |
| Serum C3, g/L ^a^ | 1.10±0.32 | 1.11±0.54 | 0.697 |
| Serum C4, g/L ^a^ | 0.27±0.10 | 0.27±0.13 | 0.739 |
| Serum IgG, g/L ^a^ | 9.87±3.92 | 10.62±3.71 | 0.010^*^ |
| Serum IgA, g/L ^a^ | 2.98±1.22 | 2.95±1.21 | 0.633 |
| AKI, n (%) ^c^ | 22(3.7) | 21(3.2) | 0.757 |
| CKD, n (%) ^c^ |  |  | 0.433 |
| CKD1 | 310(52.1) | 334(51.6) |  |
| CKD2 | 124(20.8) | 131(20.2) |  |
| CKD3 | 80(13.4) | 104(16.1) |  |
| CKD4 | 35(5.9) | 25(3.9) |  |
| CKD5 | 24(4.0) | 32(4.9) |  |

MAP, mean arterial pressure; Hb, hemoglobin; Cr, creatinine; eGFR, estimated glomerular filtration rate; UA, uric acid; BUN, blood urea nitrogen; Alb, albumin; AKI, acute kidney injury; CKD, chronic kidney disease. ^a^ normal distribution was expressed as mean ± standard deviation and compared using the *t* test. ^b^ non-parametric variables were expressed as medians (interquartile ranges) and compared using the Mann–Whitney U test. ^c^ categorical variables were expressed as frequency (percentage) and compared using the chi-square test. ^*^*p*<0.05; ^**^*p*<0.001.

**Table S2.** Pathological characteristics of IgAN patients with and without crescents

|  | Crescent group  (n=595) | Non-crescent group  (n=647) | *p* Value |
| --- | --- | --- | --- |
| IgA, n (%) ^c^ |  |  | 0.528 |
| 1~2+ | 152(25.5) | 183(28.3) |  |
| 3+ | 418(70.3) | 440(68.0) |  |
| 4+ | 25(4.2) | 24(3.7) |  |
| IgG, n (%) ^c^ |  |  | 0.124 |
| Negative | 477(80.2) | 488(75.4) |  |
| 1+ | 97(16.3) | 134(20.7) |  |
| 2~3+ | 21(3.5) | 25(3.9) |  |
| IgM, n (%) ^c^ |  |  | 0.650 |
| Negative | 135(22.7) | 139(21.5) |  |
| 1+ | 334(56.1) | 380(58.7) |  |
| 2~3+ | 126(21.2) | 128(19.8) |  |
| C3, n (%) ^c^ |  |  | <0.001^**^ |
| Negative ~1+ | 129(21.7) | 221(34.2) |  |
| 2+ | 298(50.1) | 276(42.7) |  |
| 3~4+ | 168(28.2) | 150(23.2) |  |
| M1, n (%) ^c^ | 291(48.9) | 37(5.7) | <0.001^**^ |
| E1, n (%) ^c^ | 190(31.9) | 28(4.3) | <0.001^**^ |
| S1, n (%) ^c^ | 324(54.5) | 65(10.0) | <0.001^**^ |
| T1, n (%) ^c^ | 57(9.6) | 9(1.4) | <0.001^**^ |
| T2, n (%) ^c^ | 30(5.0) | 8(1.2) | <0.001^**^ |
| Global glomerulosclerosis, % ^b^ | 0(0-12.50) | 0(0-11.10) | 0.156 |
| Interstitial inflammation, n (%) ^c^ |  |  | <0.001^**^ |
| Negative | 140(23.5) | 348(53.8) |  |
| Mild | 391(65.7) | 131(20.2) |  |
| Moderate | 33(5.5) | 152(23.5) |  |
| Severe | 31(5.2) | 16(2.5) |  |

The results of IgA, IgG, IgM and C3 were manifestations of immunofluorescence. Cellular/ fibrous/fibrocellular crescents were calculated according to the relative ratio. ^b^ non-parametric variables were expressed as medians (interquartile ranges) and compared using the Mann–Whitney U test. ^c^ categorical variables were expressed as frequency (percentage) and compared using the chi-square test. ^*^*p*<0.05; ^**^*p*<0.001.

**Table S3.** Univariate analysis of clinical and pathological factors for the combined events

| Parameters | ESKD | | | Combined events | | |
| --- | --- | --- | --- | --- | --- | --- |
|  | HR | 95%CI | *p* Value | HR | 95%CI | *p* Value |
| Gender | 0.634 | 0.388-1.036 | 0.069 | 0.693 | 0.467-1.028 | 0.068 |
| Age, year | 1.001 | 0.980-1.023 | 0.897 | 1.018 | 1.002-1.035 | 0.029^*^ |
| MAP | 1.028 | 1.011-1.045 | 0.001^*^ | 1.032 | 1.018-1.047 | <0.001^**^ |
| Cr, μmol/L | 1.004 | 1.003-1.005 | <0.001^**^ | 1.002 | 1.001-1.003 | <0.001^**^ |
| eGFR, ml/min/1.73 m^2^ | 0.977 | 0.970-0.984 | <0.001^**^ | 0.988 | 0.983-0.993 | <0.001^**^ |
| UA, μmol/L | 1.004 | 1.002-1.006 | <0.001^**^ | 1.004 | 1.002-1.005 | <0.001^**^ |
| BUN, mmol/L | 1.282 | 1.222-1.346 | <0.001^**^ | 1.317 | 1.259-1.378 | <0.001^**^ |
| Hb, g/L | 0.967 | 0.966-0.979 | <0.001^**^ | 0.971 | 0.961-0.980 | <0.001^**^ |
| Alb, g/L | 1.004 | 0.995-1.013 | 0.382 | 1 | 0.992-1.009 | 0.992 |
| Gross hematuria | 0.393 | 0.194-0.794 | 0.009^*^ | 0.644 | 0.394-1.053 | 0.079 |
| 24h urinary protein, g/24h | 1.203 | 1.137-1.272 | 0.001^*^ | 1.164 | 1.018-1.331 | 0.026^*^ |
| Urinary RBC |  |  |  |  |  |  |
| Negative | Reference | | | Reference | | |
| 1+ | 0.597 | 0.207-1.722 | 0.340 | 0.482 | 0.167-1.393 | 0.178 |
| 2+ | 1.138 | 0.489-2.649 | 0.764 | 0.33 | 0.123-0.885 | 0.028^*^ |
| 3+ | 1.468 | 1.468-0.676 | 0.332 | 0.694 | 0.293-1.644 | 0.406 |
| Serum C3, g/L | 0.614 | 0.247-1.524 | 0.293 | 0.651 | 0.277-1.530 | 0.324 |
| Serum C4, g/L | 0.985 | 0.076-12.798 | 0.991 | 4.627 | 0.506-42.351 | 0.175 |
| Serum IgG, g/L | 0.947 | 0.876-1.023 | 0.166 | 0.977 | 0.904-1.057 | 0.568 |
| Serum IgA, g/L | 1.224 | 0.982-1.525 | 0.072 | 1.208 | 1.009-1.446 | 0.040^*^ |
| Proportions of crescents |  |  |  |  |  |  |
| None | Reference | | | Reference | | |
| <10% | 2.627 | 1.343-5.346 | 0.005^*^ | 2.262 | 1.312-3.898 | 0.003^*^ |
| 10~24% | 2.087 | 1.093-3.985 | 0.026^*^ | 1.529 | 0.906-2.582 | 0.112 |
| 25~49% | 2.269 | 0.950-5.418 | 0.065 | 2.48 | 1.327-4.632 | 0.004^*^ |
| ≥50% | 6.634 | 2.634-16.639 | <0.001^**^ | 3.781 | 1.585-9.019 | 0.003^*^ |
| M1(M0/M1) | 2.539 | 1.487-4.333 | 0.001^*^ | 2.041 | 1.340-3.110 | 0.001^*^ |
| E1(E0/E1) | 1.570 | 0.936-2.632 | 0.087 | 1.251 | 0.814-1.923 | 0.307 |
| S1(S0/S1) | 1.922 | 1.117-3.305 | 0.018^*^ | 1.871 | 1.208-2.899 | 0.005^*^ |
| T1(T0/T1/T2) | 4.881 | 2.848-8.364 | <0.001^**^ | 7.312 | 4.561-11.723 | <0.001^**^ |
| T2(T0/T1/T2) | 8.138 | 4.789-13.827 | <0.001^**^ | 9.038 | 5.551-14.718 | <0.001^**^ |
| Global glomerulosclerosis, % | 1.03 | 1.025-1.051 | <0.001^**^ | 1.037 | 1.026-1.047 | <0.001^**^ |
| Interstitial inflammation, % |  |  |  |  |  |  |
| Negative | Reference | | | Reference | | |
| Mild | 0.982 | 0.559-1.887 | 0.933 | 1.153 | 0.712-1.866 | 0.564 |
| Moderate | 1.458 | 0.745-3.202 | 0.242 | 1.668 | 0.927-3.001 | 0.088 |
| Severe | 5.136 | 2.482-10.641 | <0.001^**^ | 4.177 | 2.267-7.696 | <0.001^**^ |
| Cellular crescent, % | 1.606 | 0.798-3.232 | 0.185 | 1.504 | 0.855-2.646 | 0.157 |
| Fibrous crescent, % | 2.167 | 1.182-3.974 | 0.012^*^ | 1.773 | 1.062-2.958 | 0.028^*^ |
| Fibro-cellular, % | 1.441 | 0.743-2.793 | 0.279 | 1.336 | 0.778-2.294 | 0.293 |

MAP, mean arterial pressure; Cr, creatinine. ^*^ *p*<0.05; ^**^*p*<0.001.

**Table S4.** Comparison of baseline characteristics of IgAN patients in this discovery cohort and validation cohort

|  | Discovery cohort  (n=895) | Validation cohort (n=346) | *p* Value |
| --- | --- | --- | --- |
| Gender, Male/Female ^c^ | 393/502 | 136/210 | 0.141 |
| Age, Year ^a^ | 34.56±11.99 | 36.34±11.97 | 0.019 |
| Hypertension,%^c^ | 246(27.5) | 104(30.1) | 0.03 |
| SCr, μmol/L ^b^ | 120.27±111.45 | 132.39±118.15 | <0.001^**^ |
| eGFR, ml/min/1.73m^2 b^ | 87.28±39.61 | 72.75±38.39 | <0.001^**^ |
| Alb, g/L ^b^ | 39.30±18.12 | 36.68±7.63 | <0.001^**^ |
| Gross hematuria, n(%)^c^ | 262(29.3) | 109(31.5) | 0.442 |
| 24h urinary protein, g/24h ^b^ | 1.37(0.75-2.54) | 1.02(0-2.80) | <0.001^**^ |
| Global glomerulosclerosis, % ^a^ | 7.90±13.02 | 15.92±18.51 | <0.001^**^ |
| Interstitial inflammation, n (%) ^c^ |  |  | <0.001^**^ |
| Negative | 228(25.5) | 116(33.5) |  |
| Mild | 549(61.3) | 150(43.4) |  |
| Moderate | 78(8.7) | 59(17.1) |  |
| Severe | 40(4.5) | 21(6.0) |  |
| Proportions of crescents^a^ | 8.00±12.41 | 20.81±25.20 |  |

Hb, hemoglobin; Cr, creatinine; eGFR, estimated glomerular filtration rate; Alb, albumin; ^a^ normal distribution was expressed as mean ± standard deviation and compared using the *t* test. ^b^ non-parametric variables were expressed as medians (interquartile ranges) and compared using the Mann–Whitney U test. ^c^ categorical variables were expressed as frequency (percentage) and compared using the chi-square test. ^*^*p*<0.05; ^**^*p*<0.001.

 **Figure S1.** Distribution of the percentage of glomeruli with crescents in biopsies with any crescents.
